# Loneliness and risky behaviours among mobile fishers in Elmina, Ghana: a convergent parallel mixed-method study

**DOI:** 10.1186/s12889-024-19243-w

**Published:** 2024-06-30

**Authors:** Sylvester Kyei-Gyamfi, Frank Kyei-Arthur

**Affiliations:** 1 Department of Children, Ministry of Gender, Children and Social Protection, Accra, Ghana; 2 Department of Environment and Public Health, University of Environment and Sustainable Development, Somanya, Ghana

**Keywords:** Fishers, Loneliness, Risky behaviours, Coping strategies, Ghana

## Abstract

**Background:**

Loneliness affects individuals of all age groups, and mobile fishers are susceptible to loneliness due to the nature of their occupation. However, there is no study examining loneliness and risky behaviours among fishers in Ghana. Therefore, the purpose of this study was to examine fishers’ mobility history, prevalence of loneliness, predictors of loneliness, effects of loneliness on fishers, coping strategies to address loneliness, and prevalence of risky behaviour among fishers in Elmina, Ghana.

**Methods:**

This is a convergent parallel mixed-method study involving 385 fishers in Elmina. A questionnaire and interview guides were used to collect data from respondents. Descriptive statistics, Pearson’s chi-square and Fisher exact tests, and binary logistic regression were used to analyse the quantitative data, while the qualitative data were analysed thematically.

**Results:**

From the quantitative findings, most fishers were mobile (54.5%) and travelled alone (45.7%). Approximately 83% of the fishers experienced loneliness. Male fishers (AOR = 0.049; 95% CI = 0.003–0.741; p-value = 0.030), fishers affiliated with the African Traditionalist religion (AOR = 0.043; 95% CI = 0.002–0.846; p-value = 0.038), and fishers who travelled with their working colleagues (AOR = 0.002; 95% CI = 0.000-0.023; p-value = ≤ 0.001), were less likely to be experience loneliness. Feeling bored, isolated and worried/anxious were the main perceived effects of loneliness. Alcohol consumption and finding a companion to spend time with were the main strategies fishers used to cope with their loneliness. Most male fishers consumed alcohol (92.5%; p-value = ≤ 0.001) and spent time with companions (73.5%; p-value = ≤ 0.001) to cope with their loneliness. The quantitative and qualitative findings showed that fishers engaged in risky behaviours (excessive alcohol consumption, casual sex, and smoking marijuana and tobacco). From the quantitative findings, more male fishers engaged in excessive alcohol consumption (97.6% vs. 74.5%; p-value = ≤ 0.001), casual sex (88.2% vs. 61.7%, p-value = ≤ 0.001), smoking marijuana (43.0% vs. 13.0%, p-value = ≤ 0.001) and tobacco (49.4% vs. 19.1%; p-value = 0.001) than female fishers.

**Conclusions:**

Loneliness and risky behaviours were common among fishers. Therefore, there is an urgent need to design interventions to help reduce loneliness and risky behaviour among fishers.

## Background

Loneliness is a subjective experience characterised by a perceived absence or deprivation of companionship, which arises from a disparity between an individual’s actual and desired state of social connections [1–4]. According to Hawkley [5], loneliness is a feeling that arises when an individual perceives their social connections to be inadequate, as they would like them to be. Globally, 33% of persons aged 16–74 experienced loneliness as of 2021 [6]. Persons in Brazil reported the highest prevalence (50%) of loneliness, while those from the Netherlands reported the lowest prevalence (15%) of loneliness [6]. In South Africa, 40% of persons reported feeling loneliness.

Loneliness, despite its unpleasantness, is a ubiquitous human feeling that individuals of all age groups encounter at some stage in their lives [7, 8]. Nevertheless, persistent experience of loneliness has detrimental effects on health outcomes and wellbeing, such as sleep disturbance [9], coronary heart disease and stroke [10], depression [11, 12], anxiety [13] and poorer subjective wellbeing [14, 15].

Loneliness occurs in diverse contexts, including educational, occupational, and community contexts [3, 14, 16]. For instance, a meta-analysis by Bryan et al. [16] revealed that loneliness is associated with the work environment. Bryan et al. [16] also reported that loneliness in the workplace decreases job satisfaction and performance while increasing burnout. Erdil and Ertosun’s [17] study in Turkey found that employees’ loneliness at the workplace affects job-related wellbeing through two mechanisms. On the one hand, employees’ loneliness increases negative job-related wellbeing. On the other hand, employees’ loneliness decreases positive job-related wellbeing. Also, Moens et al.’s [18] study in Belgium found that temporarily contracted employees experience more loneliness at the workplace compared to permanently contracted employees. Also, Moens et al. [18] reported that employees’ loneliness moderates the relationship between working temporarily and job satisfaction.

Occupations characterised by extended working hours and frequent travel can exert pressure on social interactions and contribute to feelings of loneliness. For example, mobile fishers have been recognised as being susceptible to feelings of loneliness due to the inherent demands of their occupation, which often require prolonged absences from their homes while they operate at multiple fishing communities [19–21]. Most of the fishers who relocate do so without their families or partners, which causes them to become detached from their social and family connections [19].

Studies have found a link between loneliness and risky behaviour, such as substance use [22, 23]. For instance, a study by Stickley et al. [24] among Russian and U.S. adolescents revealed that loneliness increased adolescents’ odds of alcohol consumption, use of marijuana and other illicit drugs, and pregnancy. Similarly, a study by Gutkind [22] in the US among adult substance users revealed that respondents with moderate or severe loneliness had greater odds of alcohol and cannabis use.

Although studies have been conducted on loneliness in Ghana, these studies have focused on adolescents [25, 26] and older adults [27–29]. Additionally, studies have shown that fishers engage in risky behaviours [30–32]. Kyei-Arthur and Kyei-Gyamfi’s [32] study in Ghana among fishers revealed that approximately 59% of fishers engaged in alcohol consumption. Additionally, Seeley et al.’s [33] case study in Uganda revealed that fishers had multiple sexual partners. Zafar et al.’s study among fishers in Karachi revealed that approximately 92% were engaged in unsafe sexual practices, such as non-condom use and multiple sexual partners.

However, to the best of our knowledge, no study has examined loneliness and risky behaviours among fishers in Ghana. To bridge this knowledge gap, this study examined fishers’ mobility history, prevalence of loneliness, predictors of loneliness, effects of loneliness on fishers, coping strategies to address loneliness, and prevalence of risky behaviour among fishers in Elmina, Ghana. The investigation of loneliness and risky behaviours among fishers will provide policymakers with insights into the impact of fishers’ occupational setting on their experiences of loneliness and engagement in risky behaviours. This understanding can inform strategies aimed at mitigating loneliness and risky behaviours among fishers. This study had two hypotheses: (a) female fishers are less likely to experience loneliness compared to male fishers, and (b) fishers who travelled with their working colleagues are less likely to experience loneliness compared to fishers who travelled alone.

## Methods

### Study design and sampling procedure

This study used a convergent parallel mixed-method design. A convergent parallel mixed-method design was used because the researchers concurrently collected qualitative and quantitative data. The design allows researchers to compare, interrelate or validate findings from qualitative and quantitative data [34]. In this study, qualitative and quantitative data were interrelated to understand the phenomenon of loneliness and risky behaviours among fishers in Elmina, Ghana.

Since the population size of the fishers in the study area is unknown, the sample size for the quantitative study was determined using the following formula:

$$n=\frac{{\left(Z score\right)}^{2}*StdDev\left(1-StdDev\right) }{{\left(Margin \ of\ error\right)}^{2}}$$

where n = sample size, Z score = 1.96 for a 95% confidence interval, standard deviation (StdDev) = 0.5, and margin of error = 0.05.

$$n=\frac{{\left(1.96\right)}^{2}*0.5\left(1-0.5\right)}{{\left(0.05\right)}^{2}}$$

$$n=384.16$$

The sample size calculation resulted in 384.16 participants, which was rounded up to 385 participants who were administered with the questionnaire for the quantitative data. After compiling the list of fishers for ten identified fisher associations, a population of 690 participants was determined through the listing process. To guarantee that the fishers in each of the ten associations had an equal chance of being picked for inclusion in the study, a simple random selection by proportionate allocation was used. The following formula was used to determine the sample size of 385:

$$\text{F}\text{i}\text{s}\text{h}\text{e}\text{r\ } \text{A}\text{s}\text{s}\text{o}\text{c}\text{i}\text{a}\text{t}\text{i}\text{o}\text{n} \left(\text{y}\right) =$$

$$\frac{n \left(sample\ size\right)}{N \left(total \ population\right)} \times \text{N}1\left(\text{m}\text{e}\text{m}\text{b}\text{e}\text{r}\text{s} \ \text{i}\text{n}\ \text{e}\text{a}\text{c}\text{h} \ \text{a}\text{s}\text{s}\text{o}\text{c}\text{i}\text{a}\text{t}\text{i}\text{o}\text{n}\right)$$

The names of the 690 fishers were written on pieces of paper and put into ten containers, each representing one fisher association. The names of the fishers were drawn at random from the containers, and the expected sample size was chosen for association. Research assistants administered questionnaires to respondents at places respondents felt comfortable being interviewed, such as their homes and community centres. On average, the administration of a questionnaire lasted for 35 min.

For the qualitative data, focus group discussions (FGDs) and key informant interviews (KIIs) were conducted using purposive sampling. Purposive sampling was used because the researchers were interested in selecting participants who could provide the relevant information to achieve the research objectives [35]. Regarding the FGDs, separate sessions were conducted - one with male fishers and another with female fishers. From each of the ten fishers’ associations, one male and one female fisher were chosen to participate in the FGDs. As a result, each male and female FGD included a representative from all ten fishers’ associations. This approach was adopted to ensure comprehensive coverage of viewpoints from all fishers’ associations. Also, the fishers selected for the FGDs were not interviewed for the quantitative data.

The FGDs were conducted in community centres since participants perceived them as convenient. On average, the FGDs lasted for 80 min. Research assistants were recruited and trained to collect the quantitative and qualitative data. Two research assistants conducted the FGDs. One research assistant was the moderator of the discussion, while the other was the notetaker.

For the KIIs, thirty KIIs were purposively selected and interviewed. The key informants interviewed included officials of the KEEA Municipal Assembly, Department of Fisheries, Ghana AIDS Commission, and members of the fisheries associations. More details about the sampling procedure can be found in previous studies [32]. Research assistants conducted KIIs at places key informants perceived as convenient, such as their offices, conference rooms, and residences. On average, the KIIs lasted for 45 min.

### Study setting

This study was conducted in Elmina in the KEEA Municipal Assembly in the Central Region of Ghana. Elmina is in the Central Region of Ghana on the country’s South Coast, 12 km West of Cape Coast, the regional capital of the Central Region, on the Atlantic Ocean coast. The KEEA Municipal Assembly was detached from the Cape Coast Metropolis in 1988 and upgraded to a municipality in 2008. The municipality is bordered on the South by the Atlantic Ocean (Gulf of Guinea), on the East by the Cape Coast Metropolis, on the North by the Twifo-Hemang-Lower Denkyira District, and on the West by the Mpohor-Wassa East District [36].

The KEEA Municipal Assembly has a total population of 166,017, an average household size of 3.3 and a population density of 354.7 persons per square kilometer as of 2021 [37].

### Outcome and measurement of variables

#### Dependent variable

Loneliness was measured by asking fishers, ‘Have you ever experienced loneliness in any of your travels in the last 12 months preceding the study?’, and the responses were ‘yes’ and ‘no’. The question “Have you ever experienced loneliness in any of your travels in the last 12 months preceding the study?” was developed by the first author. The measurement of loneliness is valid since the question used to loneliness among fishers measured loneliness among fishers 12 months before the survey. Also, during the training of research assistants, they were trained to ask questions, including the question on loneliness, as it is written to ensure consistency in how the questions are asked during the data collection. This helps ensure the content validity of the questions. Also, research assistants asking respondents’ questions in the same way helps reduce variability in the responses, which improves reliability.

#### Independent variables

Risky behaviour was measured among fishers by asking, ‘In the last 12 months, have you ever engaged in excessive alcohol consumption, casual sex, tobacco or marijuana smoking during any fishing trip out of loneliness?’ The responses were ‘yes’ and ‘no’.

The socio-demographic characteristics of the fishers included in this study were sex (male and female), age (< 25, 25–44 and 45+), education (no education, middle/JHS education, secondary/vocational and higher), religion (Islam, African Traditionalist, Christianity, No religion), marital status (never married, currently married and divorced/separated/widowed), type of fishing occupation (fish catch group, post-harvest group, maintenance and repair group and porters and errand group), and persons whom respondents travelled with to other fishing communities (alone, with working colleagues and with family).

### Terms and definitions

Fisher refers to any person (male or female) aged 18 years or older involved in fishing-related activities in the Elmina fishing community. The activities include actual fish harvest, mending of fish nets, building and repairing fishing canoes/boats, fish processors, traders of fish, fishing gears, transporting of fish, and pottering of fish and fishing gear.

Mobility refers to any travel outside the study community of Elmina to engage in any fishing-related activity, which required the respondent to spend at least one night at the destination point in the last 12 months preceding the study.

A fishing community refers to a community that is substantially dependent on or substantially engaged in the harvest of fishery resources to meet social and economic needs.

### Data collection

The data for this study were collected between July and August 2017. The quantitative data covered various topics, including mobility and settlement patterns and HIV risks, HIV and related knowledge and attitudes of fishers, risky sexual behaviour, and strategies for carrying out HIV education.

The qualitative data covered similar topics, such as mobility and knowledge, attitudes and practices regarding HIV, since the qualitative data complement the quantitative data. For a participant to be included in this study, the participant must be 18 years or older, must be engaged in a fishing-related activity, must be a resident of Elmina, and be willing to participate and share his/her experience. Research assistants with at least 2 years of experience in conducting mixed-method data collection were recruited and trained on the data collection instruments. During the training, research assistants were trained on ethics in data collection. Also, they were trained on community and household entry, seeking informed consent from respondents, confidentiality and anonymity. To ensure confidentiality and privacy, respondents were interviewed at places they perceived safe and convenient. Furthermore, the data collected from respondents were anonymised to protect the identities of respondents.

Participation in the study was voluntary, and all participants who participated in this study provided written informed consent. The study was approved by the Ethics Committee for the Humanities at the University of Ghana. No incentives were given to respondents.

### Data analysis

We utilised the Statistical Package for Social Sciences (SPSS) version 25 for the input of the quantitative data. In this process, each questionnaire item was created as a variable in SPSS with its corresponding properties in the variable view of the SPSS. The responses for each item were then meticulously entered into the data view of the SPSS. To ensure accuracy and reliability, the data entry was performed by two dedicated research assistants. Furthermore, the first author conducted a thorough cross-check of the entered data, confirming that the correct responses were assigned to each variable as indicated in the questionnaire.

The quantitative data were analysed using the SPSS version 25. Frequencies and percentages were used to analyse the socio-demographic characteristics of the fishers. Pearson’s chi-square test and Fisher’s exact test were used to analyse the associations between the mobility status of the fishers and the sex of the fishers, the persons whom the fishers travelled to other fishing communities and the sex of the fishers, the reasons for travelling to other fishing communities and the sex of the fishers, the loneliness and sex of the fishers, the effects of loneliness on the fishers and sex of the fishers, loneliness and the persons whom the fishers travelled to other fishing communities, the coping strategies of the fishers and sex of the fishers, and the fishers’ engagement in risky behaviours and the sex of the fishers. Furthermore, binary logistic regression was performed to determine the predictors of loneliness among mobile fishers. All variables are statistically significant at the 95% confidence interval.

Regarding the qualitative data, all interviews were audio recorded, transcribed word-for-word, and the first author compared the transcripts with the audio recording to ensure the transcription was done word-for-word. All transcripts were imported into QSR Nvivo version 10 and analysed thematically. To obtain a general grasp of participants’ narratives, all transcripts were read. Codes were allocated to statements related to the study’s objectives. Codes that described comparable experiences were categorised into key themes. To ensure the trustworthiness of the qualitative findings, the following measures were implemented. First, the first author analysed the transcripts of the FGDs and KIIs. Afterwards, the second author with expertise in qualitative research and analysis, had a peer debriefing with the first author, where they went through all the codes and key themes and all decisions made during the peer debriefing were documented. Second, the codes and key themes were further shared with peers with expertise in qualitative research and analysis. Their feedback were used to enhance the codes and key themes.

## Results

### Socio-demographic characteristics of the fishers

Table 1 shows the socio-demographic characteristics of the fishers interviewed for the survey. More than half of the 385 fishers (51.4%) interviewed for the study were female. A little over one-fifth of fishers (22.3%) were aged less than 25 years, while about 24% were aged 45 years and older (23.6%). More than half of the fishers had attained middle or high school education (53.8%). Eight out of the 10 fishers (82.1%) were Christians, while less than one-tenth of the fishers (7.5%) had no religious affiliation. More than half of the fishers (55.6%) were currently married, while approximately 3 out of 10 (29.1%) were never married. One-fourth of the fishers (25.5%) were engaged in fish catch activities, while slightly more than one-fifth (21.6%) were engaged in porter and errand activities.

**Table 1:** Socio-demographic characteristics of the fishers

Socio-demographic characteristics	Frequency	Percentage
**Sex**		
Male	187	48.6
Female	198	51.4
**Age**		
< 25	86	22.3
25–44	208	54.0
45+	91	23.6
**Education**		
No Education	129	33.5
Middle/JHS education	207	53.8
Secondary/vocational and higher	49	12.7
**Religion**		
No religion	29	7.5
Christianity	316	82.1
Islam	21	5.5
African Traditionalist	19	4.9
**Marital Status**		
Never married	112	29.1
Currently married	214	55.6
Divorced/separated/widowed	59	15.3
**Type of fishing occupation**		
Fish Catch Group	98	25.5
Post-harvest Group	149	38.7
Maintenance and Repair Group	55	14.3
Porters and Errand Group	83	21.6

### Fishers’ mobility history and reasons for moving to other fishing communities

Table 2 presents the mobility status of the fishers disaggregated by sex. As shown in Tables 2, 54.5% of the fishers reported being mobile. A significantly greater proportion of male fishers (67.4%) were mobile than were female fishers (42.4%, p-value ≤ 0.001).

**Table 2:** Mobility status of fishers by sex

Response	Mobility status		*P*-value
	Mobile *n* (%)	Not mobile *n* (%)	
**Sex**			≤ 0.001
Male	126 (67.4)	61 (32.6)	
Female	84 (42.4)	114 (57.6)	
**Total**	**210 (54.5)**	**175 (45.5)**	

Table 3 presents the persons whom respondents travelled to other fishing communities in the last 12 months and the reasons for travelling to other fishing communities. A greater proportion of fishers (45.7%) travelled alone, while 13% travelled with their families. Significantly, more male fishers (87.5%) travelled alone, while more female fishers travelled with their families (75.0%) and with working colleagues (59.3%, p-value ≤ 0.001).

**Table 3:** Persons whom fishers travelled to other fishing communities in the last 12 months and reasons for travelling to other fishing communities by sex

Responses	Total *n* (%)	Sex of fishers		*P*-value
		Male *n* (%)	Female *n* (%)	
**Persons whom respondents travelled with to other fishing communities**				≤ 0.001
Alone	96 (45.7)	84 (87.5)	12 (12.5)	
With working colleagues	86(41.0)	35(40.7)	51(59.3)	
With family	28(13.3)	7(25.0)	21(75.0)	
**Reasons for travelling to other fishing communities**				0.009*
To buy or sell fish	92(43.8)	50(54.3)	42(45.7)	
To serve as fish porter	82(39.0)	49(59.8)	33(40.2)	
To assist family members’ fishing activity	14(6.7)	7(50.0)	7(50.0)	
To track fish in other fishing communities	13(6.2)	13(100.0)	0(0.0)	
To do repair or maintenance work on boats	9(4.3)	8 88.9)	1(11.1)	

*Fisher’s exact test

According to the qualitative data, most male fishers highlighted that it was expensive to travel with family members to engage in fishing activities in other communities. Thus, for them, it is less expensive and more convenient to embark on fishing trips alone.

*Even though I might need my children to help, hiring someone to help me work at the next fishing destination point is far more affordable. This is due to the high cost of keeping my children and spouse safe while still having to find lodging, provide for their food, and take care of sanitary issues. (FGD 1, P 3)*

Additionally, female fishers explained that they enjoyed travelling in groups to different fishing communities since it is much safer than travelling alone. One female participant stated:

*There are numerous benefits to travelling in groups. Even if we must spend the night in the market area, we are safer from sexual abuse and exploitation being together. Some young men in neighbourhoods may occasionally bother you at night if you are alone. It is also easier to find a place to sleep and split the expenses when you are in a group. (FGD 2, P 1)*

Table 3 also shows that fishers mainly travelled to other fishing communities to buy or sell fish (43.8%) and to serve as fish potters (39.0%). A greater proportion of male fishers travelled to buy or sell fish (54.3%), served as fish potters (59.8%), and repaired or did maintenance work on boats (p-value = 0.009). Additionally, only male fishers (100.0%) were engaged in tracking fish in other fishing communities. However, an equal proportion of male and female fishers (50.0%) travelled to assist family members in fishing activities.

During the FGDs, participants explained that fishers relocate to other fishing communities to access fish and earn a living. Some male participants described the following:

*In our line of work, a real fisher cannot stay in one community and say he is fishing. Such a man is not regarded as a fisher. Even our women travel to places to buy and sell fish, so we have no choice but to move to get work done. (FGD 1, P 7)*

*Because of the nature of our occupation, moving is practically necessary to obtain fish in terms of both quantity and quality. When other fishing locations are facing lean seasons, we fishers in this vicinity know where to locate our fish. We then relocate to areas where we think we can find fish. This is why we constantly look for new locations to identify and perform bumper harvests. A fish supplier loses out by remaining in one fishing town, especially during the fishing season. (FGD 1, P 1)*

The existence of fish at other fish markets and the information female fishers gathers about the mobility of male fishers to either catch fish or sell their catch influence female fishers to also move to these places. This confirms that both male and female fishers move to other communities to carry out various fishing activities.

*Some of us follow our male counterparts to other fishing areas where they go to catch fish. Fish catchers occasionally have difficulty adequately preserving their harvest; therefore, to prevent the fish from spoiling, they travel to nearby fishing villages and sell their catch at extremely low prices. One of the reasons we monitored our male counterparts was because of this, especially during the lean fishing season. (FGD 2, P 4)*

An account from a key informant from the Fisheries Department supported the mobility nature of the fishers’ work.

*It can be surprising to see fishers who do not move around to follow fish because it is assumed that they would travel to other communities to engage in fishing activities. Even individuals who do not participate in commercial fishing, such as those who repair boats, travel to other fishing communities in search of business. If they got their way, females would even go fishing, but it is against tradition in fishing towns for women to engage in actual fish-catching activities on the sea. However, women continue to travel to neighbouring fishing communities to trade fish. Fishing and travelling go hand in hand. (KII 1)*

### Prevalence of loneliness

Slightly more than eight of the ten fishers (83.3%) reported that they had ever experienced loneliness while travelling in the 12 months before the study (Table 4). There was no significant difference between male and female fishers in terms of being lonely (p-value = 0.257).

**Table 4:** Loneliness and effects of loneliness on fishers by sex

Responses	Total *n* (%)	Sex of fishers		*p*-values
		Male *n* (%)	Female *n* (%)	
**Have you ever experienced loneliness in any of your travels in the last 12 months?**				0.257
Yes	175 (83.3)	108 (61.7)	67 (38.3)	
No	35 (16.7)	18 (51.4)	17 (48.6)	
**Effects of loneliness**				≤ 0.001*
Isolated	50 (29.1)	48 (96.0)	2 (4.0)	
Depressed	15 (8.7)	9 (60.0)	6 (40.0)	
Bored	61 (35.5)	41 (67.2)	20 (32.8)	
Sense of worry/anxiety	46 (26.7)	8 (17.4)	38 (82.6)	

*Fisher’s exact test

Fishers were asked to describe how their loneliness affects them, and most fishers (35.5%) reported that they felt bored, followed by feeling isolated (29.1%), feeling worried/anxious (26.7%), and feeling depressed (8.7%) (Table 4). A greater proportion of male fishers felt isolated (96.0%), bored (67.2%) and depressed (60.0%), while a greater proportion of female fishers felt worried/anxious (82.6%, p-value ≤ 0.001).

### Association between loneliness and persons whom fishers travelled to other fishing communities

Table 5 shows the association between loneliness and persons whom fishers travelled to other fishing communities. A greater proportion of fishers who felt lonely travelled with working colleagues (94.2%) and travelled alone (92.7%). Additionally, a greater proportion of fishers (82.1%) who were not lonely travelled with their families.

**Table 5:** Association between loneliness and persons whom fishers travelled to other fishing communities in the last 12 months

	Felt lonely *n* (%)	Did not feel lonely *n* (%)	*p*-value
Persons whom respondents travelled with to other fishing communities			
Alone	89 (92.7)	7 (7.3)	≤ 0.001
With working colleagues	81 (94.2)	5 (5.8)	
With family	5 (17.9)	23 (82.1)	

From the qualitative data, FGDs with female participants found that although they have friends and work colleagues around them when they travel, they still feel lonely not having their children with them. A female FGD participant reported the following:

“*At home, my children will be making noise that keeps me shouting and making me sometimes angry at them. Now, I know that shouting is what makes me very active at home. When I am away, I get no one to shout at. Noisemaking at home is replaced with uncomfortable quietness, which makes me feel very lonely. (FGD 2, P 10)*”.

Some female fishers experienced loneliness because of changes in their normal daily home schedules while they were away from buying fish in other fishing communities.

*I must wash, clean, and make sure there is food in the house for my husband and children every morning. When I’m away from home, I feel removed from these errands that have become a routine part of my existence. When I’m not doing these tasks, I feel empty and lonely and occasionally want to return home as quickly as I can when I find the fish I’m seeking. (FGD 2, 4)*

Furthermore, male fishers mentioned that travelling alone and encountering delays in obtaining the necessary fish stock that a fisher anticipated to have in the fishing community can make it lonelier and frustrating to visit other fishing communities. One male fisher expressed the following:

*Sometimes when we go fishing, we do not want to stay long, but we end up staying longer since we are unable to secure the quantity of fish caught; we need to justify our investments. The days grow increasingly frustrating and lonely the longer we are away from home and without fish. We are bound to feel lonely if we must wait in other fishing settlements for the fish we desire. (FGD 1, P 5).*

### Predictors of loneliness

Table 6 presents the predictors of loneliness among mobile fishers. From Table 6, male fishers (AOR = 0.049; 95% CI = 0.003–0.741; p-value = 0.030), fishers affiliated with the African Traditionalist religion (AOR = 0.043; 95% CI = 0.002–0.846; p-value = 0.038), and fishers who travelled with their working colleagues (AOR = 0.002; 95% CI = 0.000-0.023; p-value = ≤ 0.001), were less likely to be experience loneliness.

**Table 6:** Predictors of loneliness among mobile fishers

	Adjusted Odds Ratio	95% Confidence Interval		*P*-value
Sex				
Male	0.049	0.003–0.741		0.030
Female (RC)				
**Age**				
< 25	0.501	0.070–3.575		0.491
25–44	0.742	0.168–3.266		0.693
45+ (RC)				
**Education**				
No Education (RC)				
Middle/JHS education	2.060	0.572–7.417		0.269
Secondary/vocational and higher	1.442	0.272–7.641		0.667
**Religion**				
No religion (RC)				
Christianity	0.239	0.021–2.669		0.245
Islam	0.115	0.004–3.288		0.206
African Traditionalist	0.043	0.002–0.846		0.038
**Marital Status**				
Never married (RC)				
Currently married	2.364	0.488–11.456		0.285
Divorced/separated/widowed	0.637	0.105–3.881		0.625
**Type of fishing occupation**				
Fish Catch Group	1.489	0.361–6.146		0.582
Post-harvest Group	1.027	0.144–7.311		0.979
Maintenance and Repair Group	1.249	0.170–9.171		0.827
Porters and Errand Group (RC)				
**Persons whom respondents travelled with to other fishing communities**				
Alone (RC)				
With working colleagues	0.001	0.000-0.014		≤ 0.001
With family	0.493	0.124–1.961		0.315

### Coping strategies to address loneliness

Table 7 presents the strategies used by fishers to cope with their loneliness. Three out of 10 fishers (30.5%) consumed alcohol to cope with their loneliness. More male fishers (92.5%) than female fishers (7.5%, p value ≤ 0.001) consumed alcohol as a coping strategy. Additionally, approximately one-fifth of the fishers (19.5%) found a companion to spend time coping with their loneliness, and this strategy was common among male fishers (73.5%). Approximately 7% of fishers took a stroll on the streets to cope with their loneliness, and this strategy was mainly used by male fishers (75.0%).

**Table 7:** How fishers cope with their loneliness

Responses	Total *n* (%)	Sex of fishers		*P*-values
		Male *n* (%)	Female *n* (%)	
**Coping strategies**				≤ 0.001*
Go out to drink at a spot	53 (30.5)	49 (92.5)	4 (7.5)	
Find a companion to spend time with	34 (19.5)	25 (73.5)	9 (26.5)	
Sleep	25 (14.4)	6 (24.0)	19 (76.0)	
Attend social events (church, group meeting etc.)	20 (11.5)	6 (30.0)	14 (70.0)	
Use phone (radio/music/chat/movies)	15 (8.6)	7 (46.7)	8 (53.3)	
Do nothing	15 (8.6)	5 (33.3)	10 (66.7)	
Take a stroll on the streets	12 (6.9)	9 (75.0)	3 (25.0)	

*Fisher’s exact test

Approximately 14%, 12% and 9% of fishers slept, attended social events and used their phones to cope with their loneliness, respectively. Sleeping, attending social events and using phones were common strategies used by female fishers (76.0%, 70.0% and 53.3%, respectively). Additionally, approximately 9% of fishers did not cope with their loneliness, and this phenomenon was common among female fishers (66.7%).

### Prevalence of risky behaviour in the last 12 months

Table 8 presents the fishers’ engagement in any risky behaviour in the 12 months before the study and the types of risky behaviours they engaged in. Seven in ten fishers (70.9%) engaged in risky behaviour 12 months before the study.

**Table 8:** Fishers’ engagement in risky behaviours

Responses	Total *n* (%)		Sex of fishers				*P*-value
			Male *n* (%)		Female *n* (%)		
	Yes	No	Yes	No	Yes	No	
**Ever engaged in risky behaviour**	124 (70.9)	51 (29.1)	77 (71.3)	31 (28.7)	47 (70.1)	20 (29.9)	0.871
Excessive alcohol consumption	118 (89.4)	14 (10.6)	83 (97.6)	2 (2.4)	35 (74.5)	12 (25.5)	≤ 0.001
Casual sex	104 (78.8)	28 (21.2)	75 (88.2)	10 (11.8)	29 (61.7)	18 (38.3)	≤ 0.001
Tobacco smoking	51 (38.6)	81 (61.4)	42 (49.4)	43 (50.6)	9 (19.1)	38 (80.9)	0.001
Marijuana smoking	43 (32.6)	89 (67.4)	37 (43.0)	49 (57.0)	6 (13.0)	40 (87.0)	≤ 0.001

In terms of the type of risky behaviour, most fishers engaged in excessive consumption of alcohol (89.4%), causal sex (78.8%), tobacco smoking (38.6%), and marijuana smoking (32.6%). More male fishers than female fishers (74.5%, 61.7%, 19.1% and 13.0%) engaged in excessive consumption of alcohol (97.6%, p-value ≤ 0.001), causal sex (88.2%, p-value ≤ 0.001), tobacco smoking (49.4%, p-value 0.001) and marijuana smoking (43.0%, p-value ≤ 0.001), respectively.

From the qualitative data, interactions with male and female fishers confirmed that some fishers engaged in risky behaviours, such as causal sex and excessive alcohol consumption, when they travelled to other fishing communities. Some fishers explained:

*While we depart Elmina for other fishing communities, some fishers engage in social interactions with new acquaintances as a means of relieving boredom and loneliness. This frequently results in the pursuit of sexual partners and engagement in casual sexual behaviours. (FGD 1, P 8)*

*We do not go fishing with our wives and girlfriends because females cannot fish. Because we do not have our women to accompany us. We look for a pub to drink when we are feeling lonely in other fishing communities. Fishers sometimes excessively consume alcohol at those pubs. At the pub, one can find a woman to keep him company. It is sometimes easy to find a partner who is similarly willing to keep you company because some of the women who feel lonely also frequently come to the pub. (FGD 1, P 10)*

## Discussion

There are limited studies on loneliness and risky behaviours among fishers in Ghana. Therefore, this study investigated fishers’ mobility history, prevalence of loneliness, effects of loneliness on fishers, coping strategies to address loneliness, and prevalence of risky behaviour among fishers in Elmina, Ghana. The findings showed that most fishers were mobile, which confirms the mobile nature of the fishers’ work [19–21, 38].

The quantitative data revealed that fishers travelled to other fishing communities for a variety of reasons, including buying or selling fish, serving as fish porters, helping family members fish, catching fish, and performing maintenance or repair on boats. Consistent with previous studies [39–42], this study revealed that there is a division of labour in the fishing value chain based on the unique roles played by each sex. A greater proportion of male fishers were motivated by buying or selling fish, serving as fisher potters, repairing or doing maintenance work on boats and tracking fish in other fishing communities, while an equal proportion of female fishers were motivated by assisting family members’ fishing activities. Studies have shown that female fishers are often engaged in harvesting and processing activities in the fishing industry [43, 44].

This study revealed that eight of ten fishers (83.3%) experienced loneliness when they moved to other fishing communities. This confirms that fishers are vulnerable to loneliness due to the nature of their work [19–21]. This finding calls for policymakers, including the Ministry of Fisheries and Aquaculture Development and fisher association leaders, to implement interventions to reduce loneliness among fishers, such as encouraging regular communication between fishers and their families and creating peer support groups among fishers, which will enhance regular communication between fishers and their families and peers.

This study contributes to the literature on the experiences of fishers by examining the predictors of loneliness, the effects of loneliness, loneliness coping strategies and risky behaviour. This study found that male fishers, fishers affiliated with the African Traditionalist religion, and fishers who travelled with their working colleagues were less likely to experience loneliness. This finding does not support the first hypothesis that female fishers are less likely to experience loneliness compared to males. Female fishers tend to spend more time with their families at home and engage in more community-oriented roles than male fishers due to male fishers spending more time at the sea and in other fishing communities. These activities may increase female fishers’ social interactions. It is therefore unexpected that male fishers have a lower likelihood of experiencing loneliness compared to female fishers. Future longitudinal quantitative studies should be conducted to confirm this finding.

This study supports the second hypothesis that fishers who travelled with their working colleagues are less likely to experience loneliness compared to fishers who travelled alone. A probable explanation is that fishers who travel with their colleagues regularly interact with these colleagues before, during and after their fishing expedition in other fishing communities which enhances their social interaction, and therefore reduces their loneliness. Studies have found social interaction with colleagues reduces loneliness [45, 46]. However, fishers who travel without their families may have limited opportunities to communicate with others, which may exacerbate their loneliness. Individuals who frequently find themselves alone are more prone to experiencing loneliness [47]. This finding highlights the need for policymakers to target fishers who travel alone for interventions to reduce loneliness among mobile fishers.

Furthermore, fishers affiliated with the African Traditionalist religion were less likely to experience loneliness than those who were not affiliated with any religious group. Followers of the African Traditionalist religion engage in community rituals and ceremonies, which strengthen their social bonds, and enhance their social interactions [48], which may reduce their likelihood of experiencing loneliness. This finding supports previous studies that religion reduces the likelihood of loneliness [49–51]. This findings contributes to the design of interventions to reduce loneliness among mobile fishers.

Regarding the effects of loneliness, fishers reported that loneliness leads to boredom, isolation, a sense of worry/anxiety, and depression. This finding supports previous studies reporting that loneliness can lead to anxiety [13] and depression [11, 12]. The experience of psychological distress by fishers, such as depression, can adversely affect their mental health and general wellbeing. These findings call for the need for policymakers to embark on public health campaigns on loneliness in fishing communities to raise awareness of the effects of loneliness on mental health and general wellbeing. Also, policymakers should provide mental health resources, such as counselling services and stress management, to fishers. The provision of these resources will equip fishers with the requisite knowledge and skills to better manage psychological distress associated with loneliness, such as boredom and isolation. Furthermore, there is a need to establish peer support groups among fishers to enhance social interactions and support each other to cope with loneliness.

Fishers used various strategies to cope with their loneliness, including alcohol consumption, spending time with a companion, sleeping, attending social events (church attendance and group meetings), using their mobile phones and doing nothing. This finding corroborates the findings of previous studies showing that religious attendance [49, 52] and alcohol consumption [32, 53] are strategies for coping with loneliness. Notably, a meta-analysis by Hom et al. [54] revealed that severe sleep problems increase the odds of perceived loneliness. Additionally, there is a bi-directional relationship between sleep and loneliness.

The study found that fishers employed various coping strategies to deal with their loneliness. Fishers used emotion-focused (go out to drink at a spot, sleep, and use phone), social coping (attend social events, and find a companion to spend time with), and avoidance coping (take a stroll on the streets, and do nothing) strategies. The use of various coping strategies has implications for the health and wellbeing of fishers. For instance, the use of emotion-focused and avoidance coping strategies are not effective in dealing with loneliness, since they provide temporary solutions. However, the use of social coping strategies by fishers can enhance their social interactions and emotional wellbeing. Public health educators should educate fishers about effective coping strategies to use to cope with their loneliness so their physical, mental and emotional wellbeing can be enhanced.

Furthermore, the study revealed that approximately 71% of the fishers engaged in risky behaviour 12 months before the study. Specifically, most fishers engaged in excessive consumption of alcohol, casual sex, and smoking tobacco and marijuana. This study confirms previous studies that found excessive consumption of alcohol [32] and sexual risk-taking behaviour (such as multiple sexual partners and non-condom use) among fishers [55]. Studies have shown that loneliness can lead to increased consumption of alcohol [56, 57]. For instance, Mohamed et al.’s [56] study among adolescents in Sweden revealed that loneliness led to increased consumption of alcohol. The study also revealed that fishers’ risky behaviours varied by sex. More male fishers engaged in excessive alcohol consumption, casual sex, and marijuana smoking, while more female fishers engaged in tobacco smoking. Based on these findings, there is an urgent need for policymakers to design sex-targeted health promotion campaigns to reduce fisher participation in these risky behaviours.

### Limitations and strengths

This study has several limitations. The study was cross-sectional, and therefore, causality could not be established. Although this cross-sectional study helps us to understand loneliness and risky behaviours among fishers, we recommend a longitudinal study examining the link between loneliness and risky behaviour, which will help us understand the nuances of the relationship between loneliness and risky behaviours among fishers. Second, because each fishing community in Ghana may have a unique setting, the conclusions of this study may not apply to all fishing communities in Ghana and beyond. Third, respondents had to recollect their engagement in risky behaviours. Since these behaviours are perceived as deviant behaviours, it is possible that those behaviours may be underreported by fishers. Despite these limitations, the study’s findings will aid policymakers in developing context-specific remedies to address loneliness and risky behaviour among Elmina’s fishers.

## Conclusion

Our study indicated that loneliness is common among fishers, and there is an urgent need to design interventions to help curb its prevalence to aid in reducing the adverse health outcomes of loneliness. The study also highlighted that both male and female fishers played multiple roles in the fishing food chain. Female fishers, fishers affiliated with no religious affiliation, and fishers who travelled alone had a higher likelihood of experiencing loneliness. These sub-groups of fishers need to be targeted in the design and implementation of interventions to reduce loneliness among fishers.

Furthermore, it was observed that fishers employed positive (e.g., sleeping and attending social events) and negative (e.g., alcohol consumption) strategies to address their loneliness. Policymakers need to assist fishers in developing positive strategies to address their loneliness. Additionally, risky behaviour was common among fishers. There is a need for health promotion campaigns to educate fishers about the adverse outcomes of their behaviour on their health and general wellbeing.

## Data Availability

The datasets generated and/or analysed during the current study are available from the corresponding at reasonable request.

## Abbreviations

FGD: Focus group discussion
JHS: Junior high school
KEEA: Komenda Edina Eguafo Abirem
KII: Key informant interview
StdDev: Standard deviation
SPSS: Statistical package for social sciences
